# The ankle cartilage cascade: incremental cartilage damage in the ankle joint

**DOI:** 10.1007/s00167-021-06755-w

**Published:** 2021-10-05

**Authors:** Jari Dahmen, Jon Karlsson, Sjoerd A. S. Stufkens, Gino M. M. J. Kerkhoffs

**Affiliations:** 1 Department of Orthopaedic Surgery, Amsterdam Movement Sciences, Amsterdam UMC, Location AMC, University of Amsterdam, Meibergdreef 9, 1105 AZ Amsterdam, The Netherlands; 2 Academic Center for Evidence Based Sports Medicine (ACES), Amsterdam UMC, Amsterdam, The Netherlands; 3 Amsterdam Collaboration for Health and Safety in Sports (ACHSS), International Olympic Committee (IOC) Research Center, Amsterdam UMC, Amsterdam, The Netherlands; 4 Department of Orthopaedics, Sahlgrenska University Hospital, Sahlgrenska Academy, Gothenburg University, Gothenburg, Sweden

**Keywords:** Osteochondral lesions of the talus, OLT, Cascade, Ankle, Cartilage

## Abstract

*Level of evidence* Editorial, Level V.

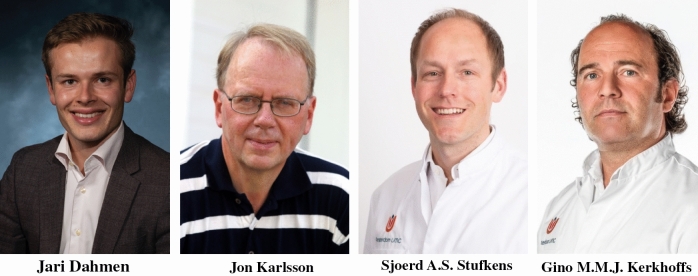



Ankle sprains remain the most frequent injuries in sporting activities [7, 8, 36]. A few decades ago, ankle sprains were assumed to be injuries that were not associated with any substantial harm. However, the last 20 years have shown that they are not as harmless as thought, as depicted in the March Issue of the year of 2016 in this Journal: *‘There is no simple lateral ankle sprain’* (Fig. 1) [13, 14]. The development of cartilage lesions following an ankle sprain is certainly food for thought, thus sparking the idea of the current Editorial. The process of cartilage degradation in the ankle following an ankle injury resembles a waterfall or cascade. Each stage sets up or “pours down” into the next stage of cartilage damage in the ankle joint.

**Fig. 1:**
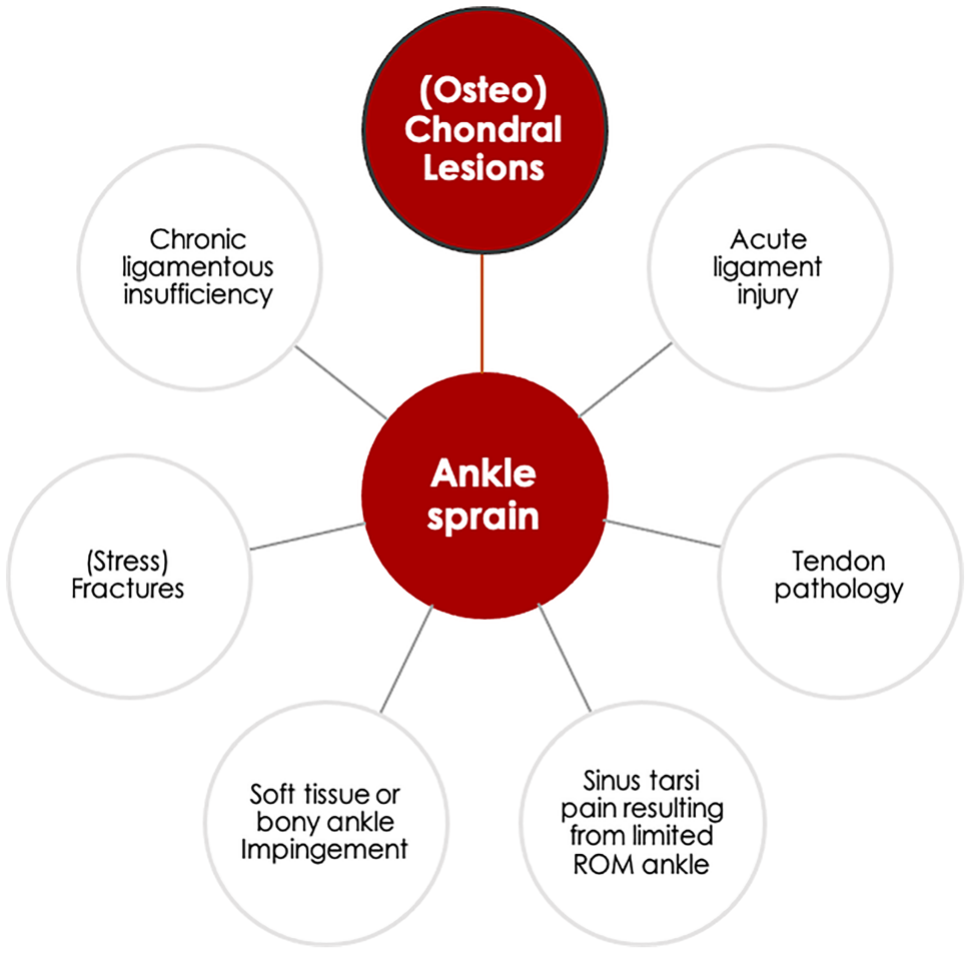
Sequential pathologies after an ankle sprain

## Asymptomatic superficial cartilage lesions

An asymptomatic cartilage lesion of the ankle, not involving any damage to the subchondral bone plate, may arise from a traumatic event, such as an ankle sprain or fracture [3]. Microscopic cartilage lesions are mostly referred to as inert, and it is believed that these lesions will not progress into an osteochondral lesion neither end-stage osteoarthritis, nor will they become symptomatic due to the fact that cartilage is avascular and a-neural tissue [16, 17, 28]. As concluded by Mankin et al. in 1974, if superficial lacerations of articular cartilage are limited in size and depth, they will ‘*neither heal nor progress to more serious disorders*’ [16, 17]. This statement can be substantiated by a recent meta-analysis that showed that although the incidence of reported osteochondral damage after ankle sprain or fracture was high (namely 45%), the proportion that may result in poor clinical outcomes (as a consequence of osteochondral damage or end-stage osteoarthritis) can be considered relatively low [5, 19, 26, 33, 37]. This finding has further been described by several studies reporting good patient-reported mid- to long-term outcomes following ankle trauma (sprains and fractures) [11, 12]. It must, however, be acknowledged that although Mankin et al. refer to the superficial lacerations as being relatively harmless or inert, it may well be the case that these superficial lesions contain microscopic cracks potentially inducive of the cartilage cascade as described and shown below [3].

## Post-traumatic cartilage cracks with damage to the subchondral bone and post-traumatic chondrocyte apoptosis

**Fig. 2:**
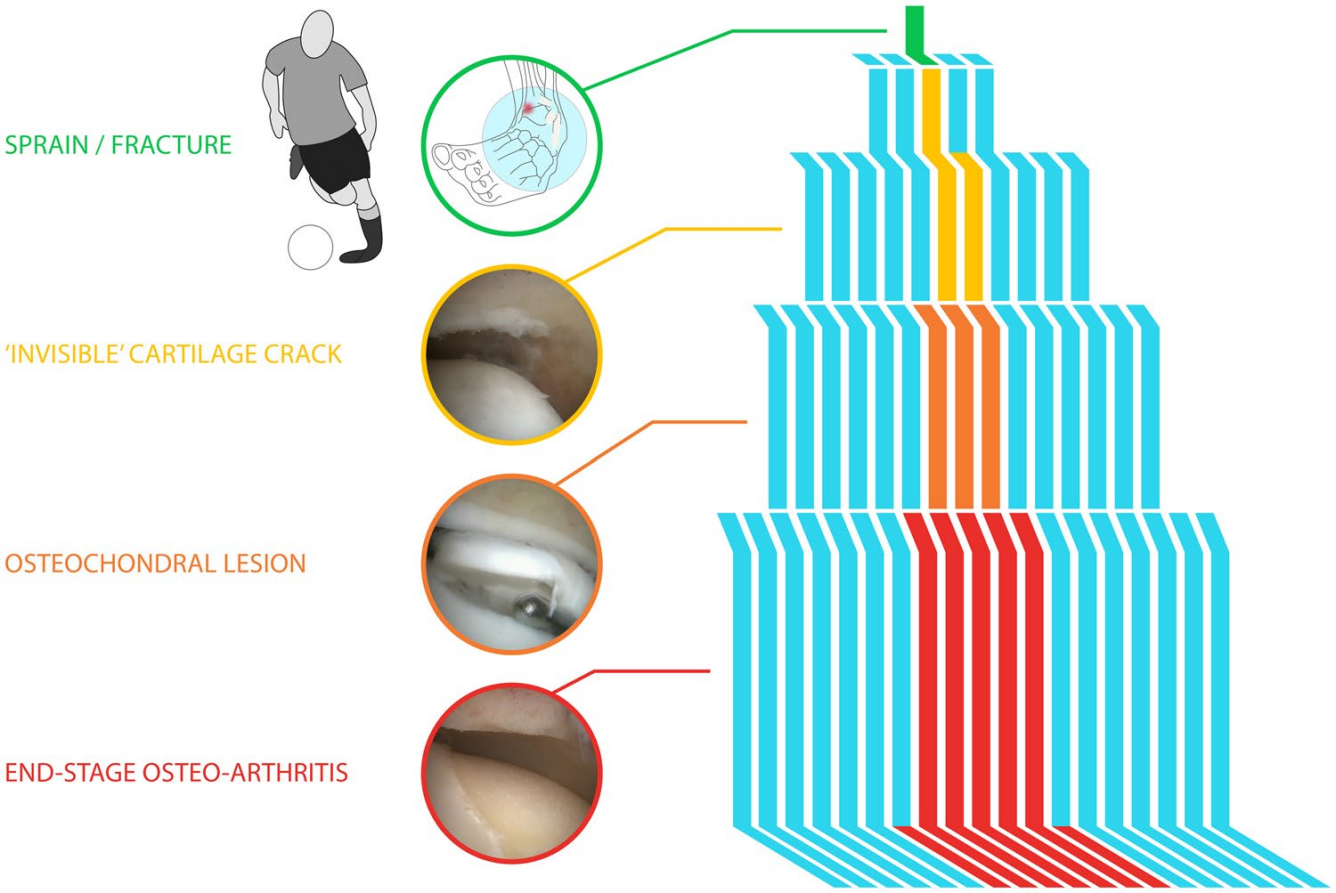
Cascade analogy, from sprain/fracture to end-stage osteo-arthritis

Cartilage lesions may become symptomatic after a traumatic event when (microscopic) cartilage cracks progress into the subchondral bone [3]. Consequently, these lesions will induce bleeding after which the lesion will either heal or fail to heal [4, 10, 16–18, 21]. The failed healing induces a cascade of events as highlighted in Fig. 2. The first stage entails a cartilage crack with subchondral bone plate damage with an insufficient repair response, that subsequently leads to an osteochondral lesion, with the potential of leading to end-stage osteo-arthritis, either localized or of the whole joint [6, 27, 32, 34]. The most interesting research question remains: why do some of the above-mentioned lesions heal while others do not?

Another entrance into the cascade is via chondrocyte apoptosis following, for example, intra-articular fractures [2, 15, 20]. A recent study by Blom et al. supports the theory of the *Incremental Cartilage Damage* (Fig. 2) in the ankle as part of the cascade [3]*.* In this study, Blom et al. investigated the presence of chondral damage after incremental single axial impact loads in ex-vivo ankle joint caprine models. After these single axial impact loads, the damage was assessed microscopically as well as through a contrast-enhanced high-resolution micro-computed tomography scan (micro-CT-scan). Furthermore, whole-joint biomechanics were assessed. Although no osteochondral damage was visible through the micro-CT-scan nor on the microscopic tests, changes were observed in the biomechanical behaviour of the joint following a single axial impact load. These changes can be a result of the alteration of the specific relative and absolute compositions and balance between proteoglycans, glycoproteins, (sulphated) glycosaminoglycans (sGAGs), and water content in the joint. Such biomechanical alternations as a result of impact load have also been observed in the knee [3, 9, 22, 25, 38].

## Large superficial or erosive cartilage lesion due to a post-traumatic status or biomechanical problem

Another potential pathophysiological pathway is a large acute superficial or erosive chronic cartilage lesion caused by trauma or an underlying biomechanical problem in or around the ankle, such as malalignment, malunion after fracture or (chronic) ligament laxity. Altered pressure distribution, edge-loading and chronic erosion of the cartilage are related to repeated microtrauma due to chronic ligament laxity and may ultimately result in a deep cartilage lesion, as a result leading to subchondral plate damage (and subsequent bleeding). A large lesion may have an insufficient repair response leading to scar tissue and fibrocartilage formation. This structure may potentially progress in size and volume, ultimately leading to degenerative changes in the entire joint. This process is different from the previously described cascade event, as its mechanism is more “delayed”. These degenerative processes have a latency time and it may take decades before symptoms become disabling [35].

## Chondrolysis after intrasynovial haemorrhage

Last, but not least, a separate entity is the cascade of chondrolysis after repeated intrasynovial hemorrhage or intra-articular bleeding. This might occur due to mechanical injury (ligament injury/laxity or capsular tears) or haemophilia. The repeated haemorrhages within the joint may lead to alterations in the cartilage matrix, more specifically leading to superficial and deep erosions where the proteoglycan concentration is low and the synthetic activity of the chondrocytes is depressed [39]. Enhanced chondrolysis can progress into damage of the subchondral bone and, thereafter, to degradation of the entire joint [16, 17]. This process might be catalyzed by chronic ligament laxity. Intra-articular bleedings may also play a role in the prior cascades where deep lesions give no guarantee of healing. It is possible that there may exist a subtle tipping point where repetitive bleeding from the subchondral bone, instead of healing the joint, erodes the cartilage and maintains a chronic synovitis.

## Take to work from the ankle cartilage cascade

Related to the description of the idea on the abovementioned cascades and the underestimated incidence rates of posttraumatic (osteo)chondral lesions, there might be different pathophysiological pathways that can induce cartilage damage and potentially lead to osteoarthritis of the ankle joint. Hence, more focus should be put on early detection and primary and secondary individualized preventive interventions to prevent cartilage damage in the earliest step of the cascade: the subclinical phase. This approach could allow for an early delay or even stop to the cartilage crack that leads to the waterfall of degeneration, and could prevent the development of osteochondral lesions and end-stage OA; two conditions with severe symptoms that affect the quality of life of the patient [1, 24]. Moreover, from a societal perspective, one can also state that this is a paramount undertaking, as we know from previous literature that end-stage OA is associated with high direct and indirect costs [12, 23]. To accomplish such goals as mentioned above, we need novel, sophisticated imaging tools [29–31] as well as innovations to cushion the joint so that we are able to diagnose and treat the cartilage damage at the early subclinical phase. [29–31]. Through presentation of the Ankle Cartilage Cascade, we hope to encourage future research to focus on the subclinical phase after ankle trauma, ultimately stalling the ongoing cascade in this phase to work towards prevention of end-stage ankle osteo-arthritis.
